# Supplementary material for: Counteranion-induced structural isomerization of phosphine-protected PdAu8 and PtAu8 clusters
Source: Commun Chem. 2023 Jun 20;6:129. doi: 10.1038/s42004-023-00929-y (PMC10281954; doi:10.1038/s42004-023-00929-y)
Supplement: Supplementary file 2 — SupplementaryInformation [file 42004_2023_929_MOESM2_ESM.pdf]

Supplementary Information

Counteranion-induced structural isomerization of phosphine-protected $PdAu_8$ and $PtAu_8$ clusters

Yu Fujiki,[1] Tomoki Matsuyama,[1] Soichi Kikkawa,[1,2] Jun Hirayama,[1,2] Hikaru Takaya,[3] Naoki Nakatani,[1] Nobuhiro Yasuda,[4] Kiyofumi Nitta,[4] Yuichi Negishi,[5] Seiji Yamazoe[1,2,6*]

[1] Department of Chemistry, Graduate School of Science, Tokyo Metropolitan University, 1-1 Minami-Osawa, Hachioji, Tokyo, 192-0397, Japan

[2] Elements Strategy Initiative for Catalysts & Batteries (ESICB), Kyoto University, 1-30 Goryo−Ohara, Nishikyo−ku, Kyoto, 615−8245, Japan

[3] Department of Life & Health Sciences, Teikyo University of Science, 2-2-1 Senjyusakuragi, Adachi-ku, Tokyo 120-0045, Japan

[4] Japan Synchrotron Radiation Research Institute (JASRI), 1-1-1 Kouto, Sayo-cho, Sayo-gun, Hyogo 679-5198, Japan

[5] Department of Applied Chemistry, Faculty of Science, Tokyo University of Science, 1-3 Kagurazaka, Shinjuku-ku, Tokyo 162-8601, Japan

[6] Precursory Research for Embryonic Science and Technology (PRESTO), Japan Science and Technology Agency (JST), Kawaguchi, Saitama 332-0012, Japan

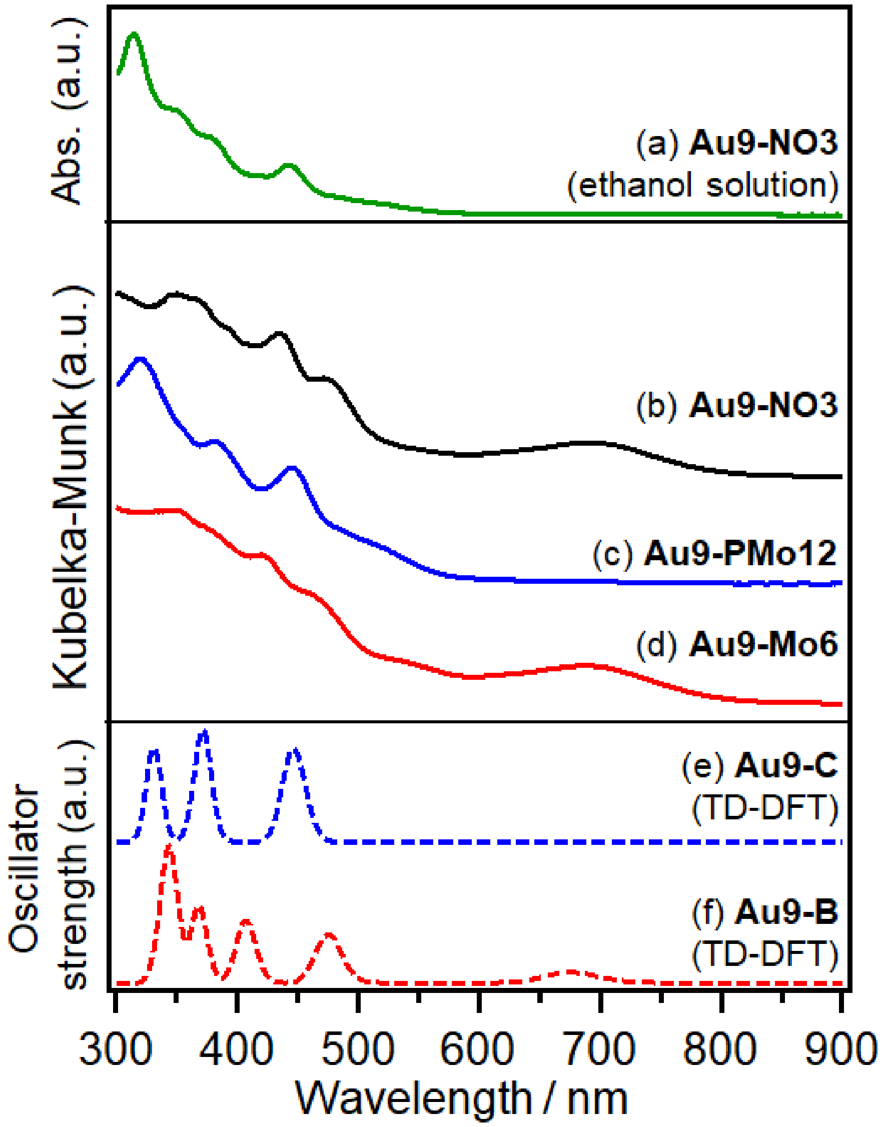

Figure S1. UV-vis-NIR spectra of (a) **Au9-NO3** ethanol solution. DR-UV-vis-NIR spectra of (b) **Au9-NO3,** (c) **Au9-PMo12**, and (d) **Au9-Mo6**, and simulated UV-vis-NIR spectra of (e) **Au9-C** and (f) **Au9-B** by TD-DFT calculation.

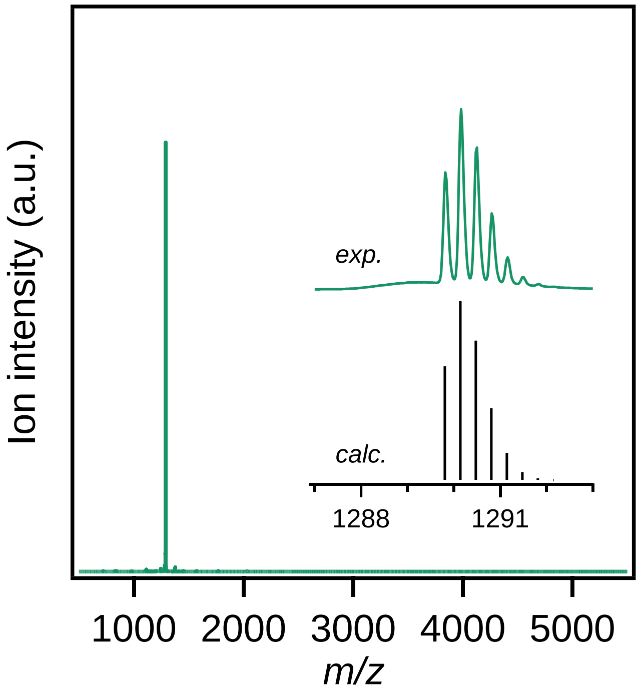

Figure S2. Positive-ion ESI mass spectra of **Au9-NO3** acetonitrile solution with experimental and calculated isotope patterns.

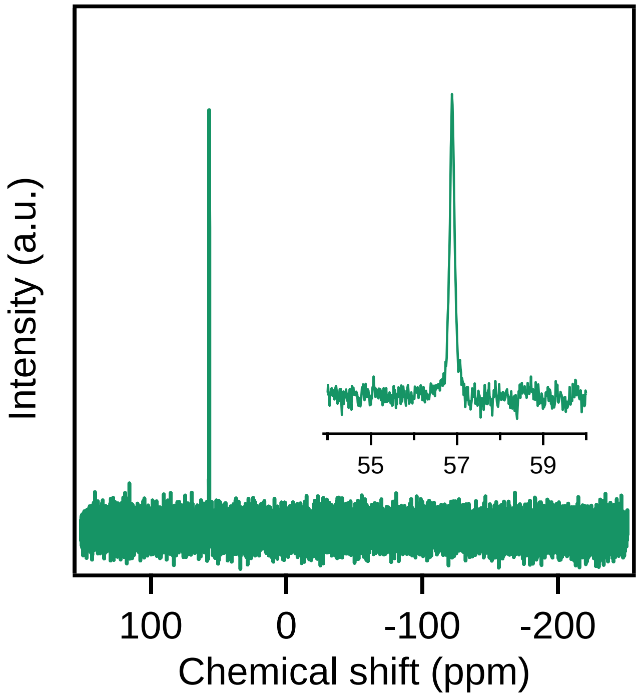

Figure S3. ^{31}P NMR spectra of **Au9-NO3** in chloroform-d.

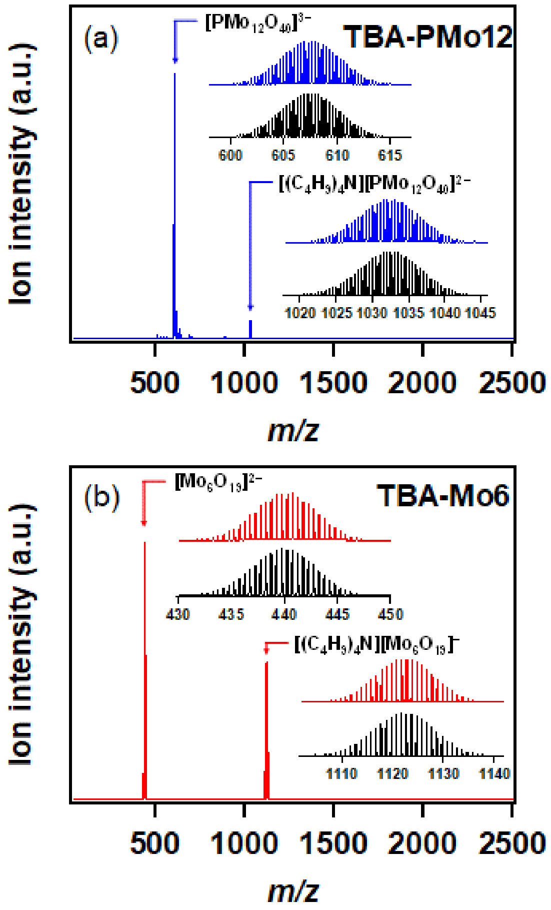

Figure S4. ESI-MS of (a) **TBA-PMo12** and (b) **TBA-Mo6**.

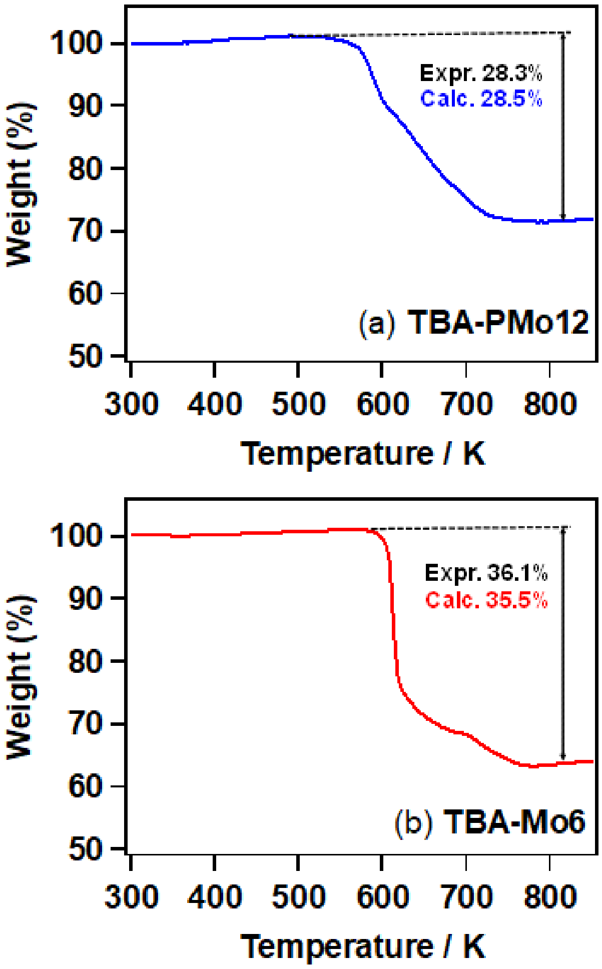

Fig. S5. TG-DTA of (a) **TBA-PMo12** and (b) **TBA-Mo6**. The weight losses in **TBA-PMo12** and **TBA-Mo6** obtained by experiments corresponded to the calculated values of $(TBA)_3[PMo_{12}O_{40}]$ and $(TBA)_2[Mo_6O_{19}]$, respectively.

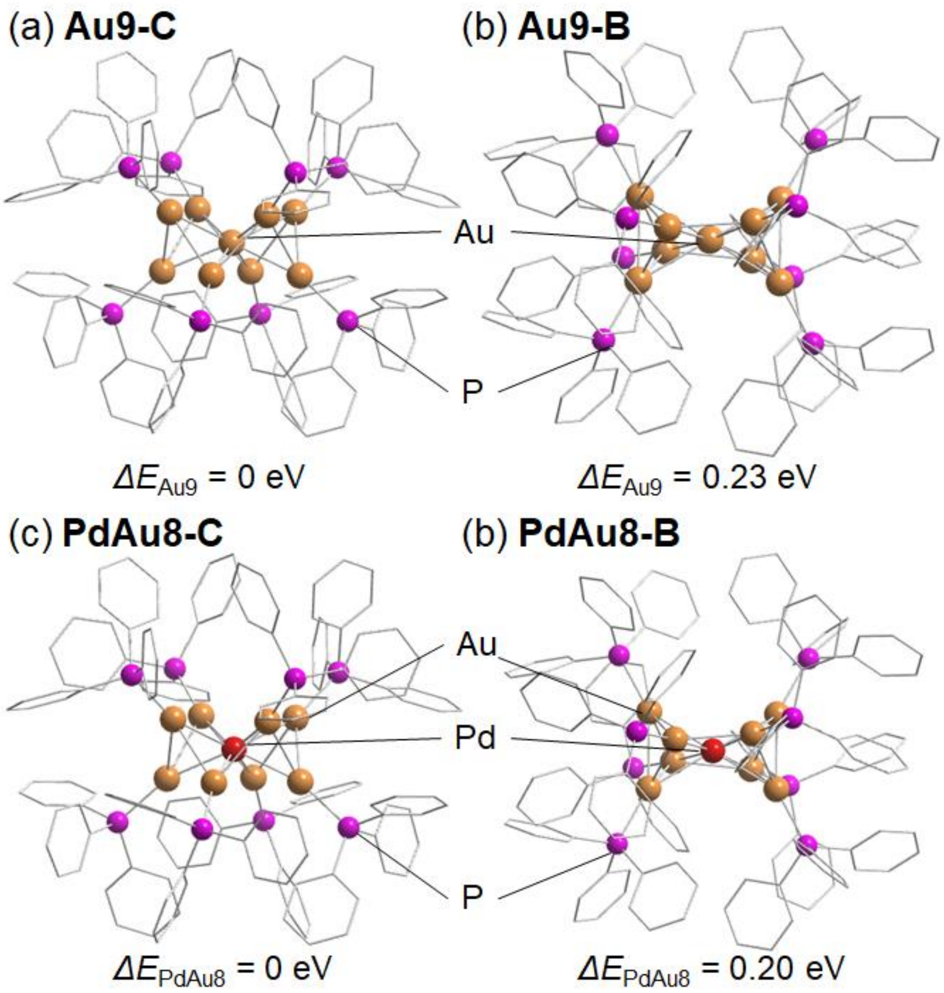

Figure S6. Optimized structures of (a) **Au9-C**, (b) **Au9-B**, (c) **PdAu8-C**, and (d) **PdAu8-B**. Color code: P (pink), Au (gold), Pd (red), and C (gray). H atoms are omitted for easy to see. The relative energies with respect to the crown-motif are shown.

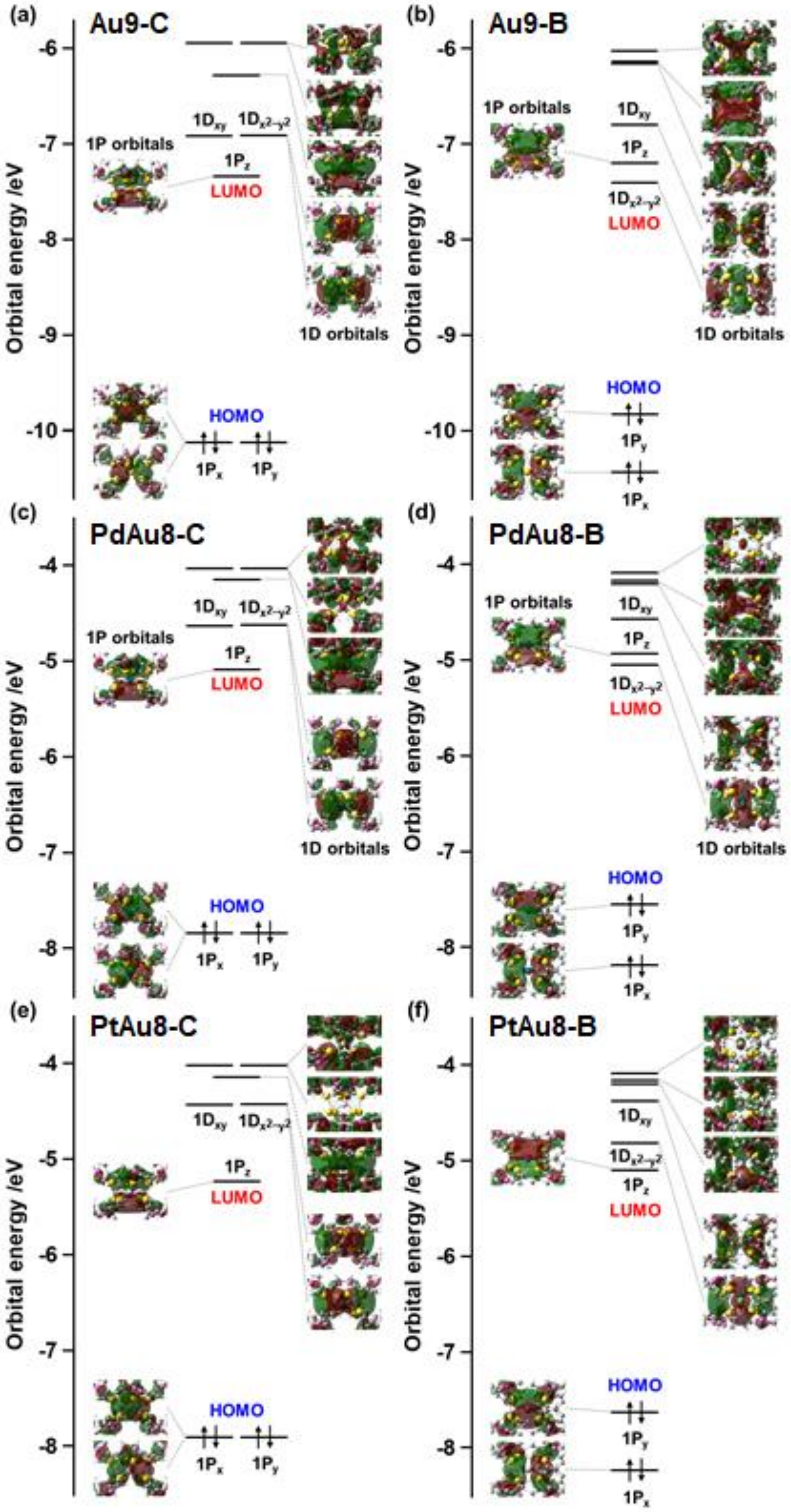

Figure S7. Molecular orbital diagrams and shapes of (a) **Au9-C**, (b) **Au9-B**, (c) **PdAu8-C**, (d) **PdAu8-B**, (e) **PtAu8-C**, and (f) **PtAu8-B**.

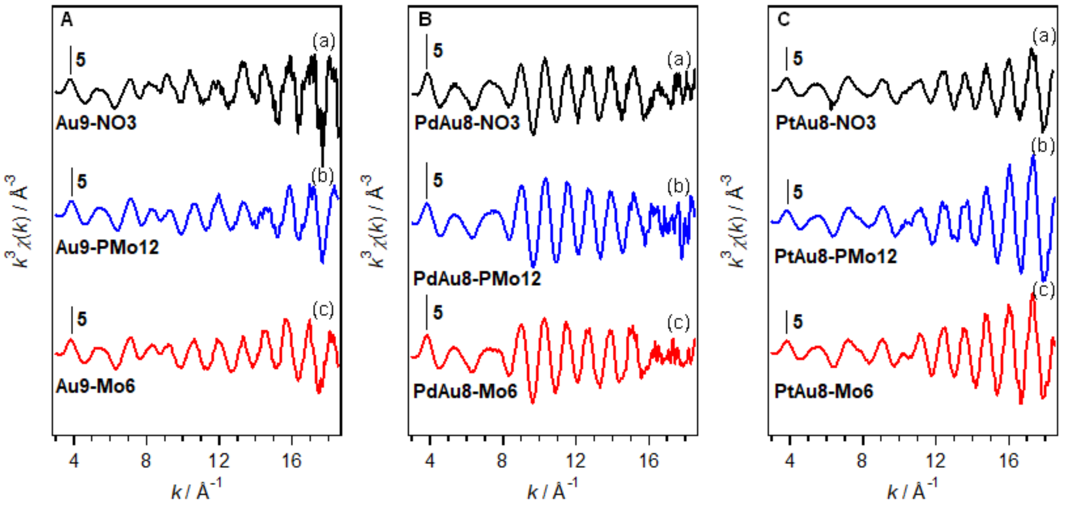

Fig. S8(A). Au L3-edge EXAFS oscillation of (a) **Au9-NO3**, (b) **Au9-PMo12**, and (c) **Au9-Mo6**. **(B)** Au L3-edge EXAFS oscillation of (a) **PdAu8-NO3**, (b) **PdAu8-PMo12**, and (c) **PdAu8-Mo6**. **(C)** Au L3-edge EXAFS oscillation of (a) **PtAu8-NO3**, (b) **PtAu8-PMo12**, and (c) **PtAu8-Mo6**. All data were collected at 10 K.

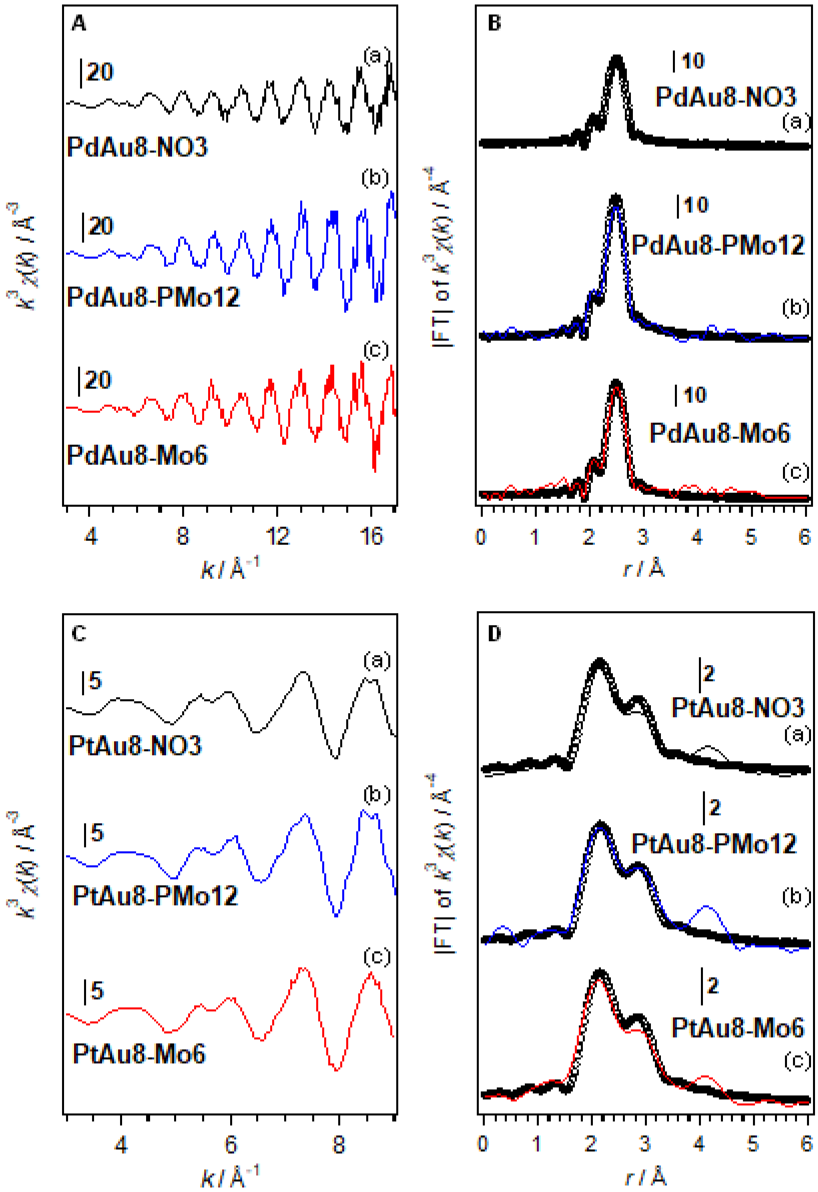

Fig. S9(A). Pd K-edge EXAFS oscillation of (a) **PdAu8-NO3**, (b) **PdAu8-PMo12**, and (c) **PdAu8-Mo6**. **(B)** Those FT-EXAFS oscillation of (a) **PdAu8-NO3**, (b) **PdAu8-PMo12**, and (c) **PdAu8-Mo6**. The circles represent the fitting curves, whose parameters and results are listed in **Table S1**. **(C)** Pt L_3-edge EXAFS oscillation of (a) **PtAu8-NO3**, (b) **PtAu8-PMo12**, and (c) **PtAu8-Mo6**. **(D)** Those FT-EXAFS oscillation of (a) **PtAu8-NO3**, (b) **PtAu8-PMo12**, and (c) **PtAu8-Mo6**. The corresponding fitting results are listed in Table S1. All data were measured at 10 K.

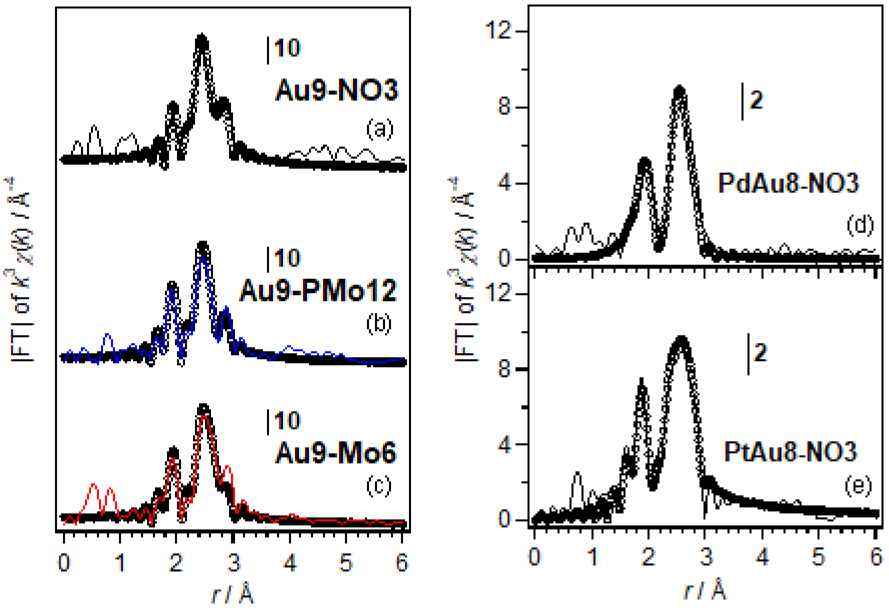

Fig. S10. Au L_3-edge FT-EXAFS spectra of (a) **Au9-NO3**, (b) **Au9-PMo12**, (c) **Au9-Mo6**, (d) **PdAu8-NO3**, and (e) **PtAu8-NO3**. The circles represent the fitting curves, whose parameters and results are listed in **Table S3**.

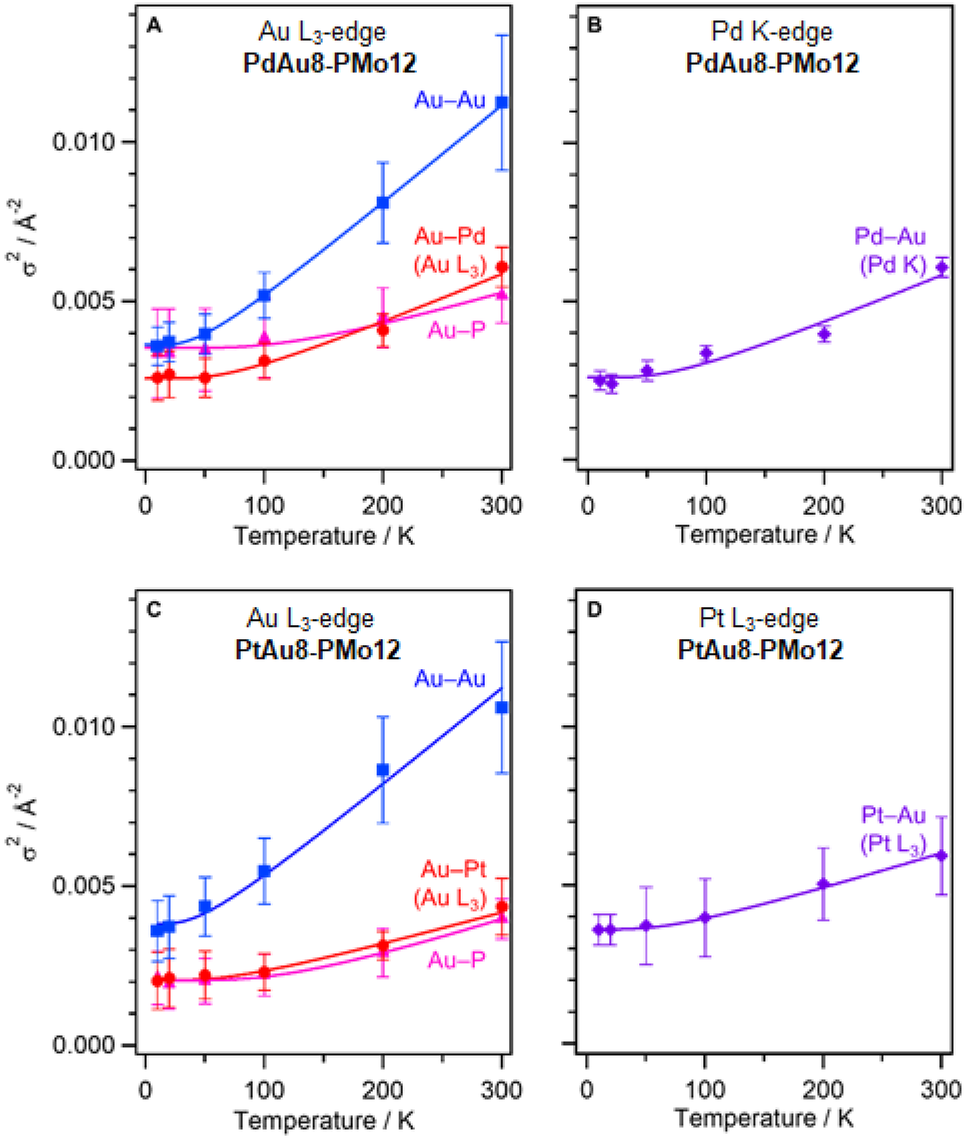

Figure S11. **(A)** Temperature dependences of DW factors of **PdAu8-PMo12** at Au L_3-edge for Au–Pd (red), Au–Au (blue), and Au–P bonds (pink). **(B)** Temperature dependences of DW factor at Pd K-edge (purple). **(C)** Temperature dependences of DW factors of **PtAu8-PMo12** at Au L_3-edge for Au–Pt (red), Au–Au (blue), and Au–P bonds (pink). **(B)** Temperature dependence of DW factor at Pt L_3-edge (purple).

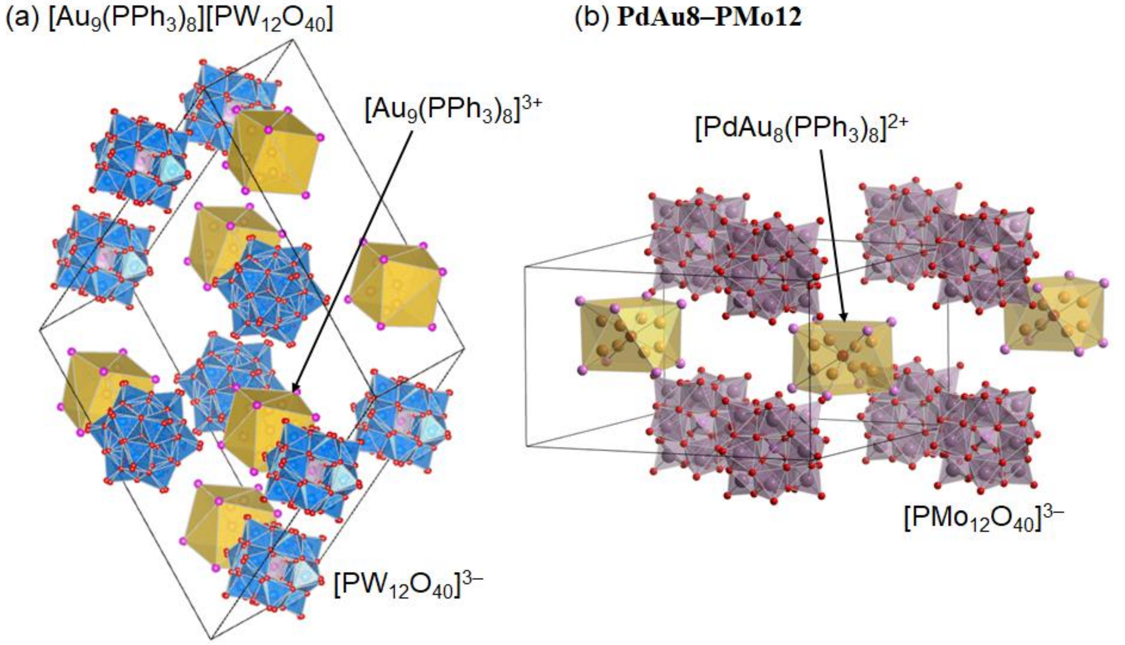

Figure. S12. Crystal packings of (a) $[Au_9(PPh_3)_8][PW_{12}O_{40}]$ (butterfly-motif **Au9-B** is surrounded by six $[PW_{12}O_{40}]^{3-}$),[1] and (b) **PdAu8-PMo12** (crown-motif **PdAu8-C** is surrounded by eight **PMo12**)[2] obtained from single crystal X-ray diffraction analysis. The C and H atoms were omitted for easy seeing.

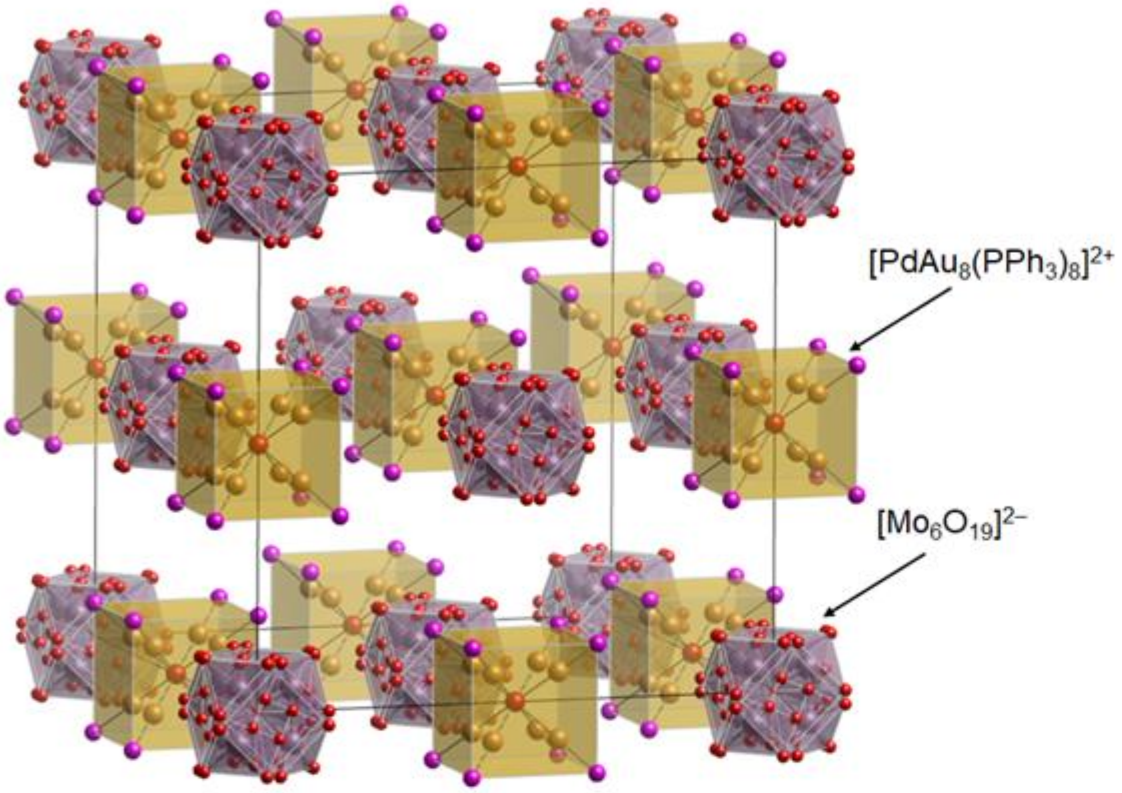

Figure. S13. Crystal packing of **PdAu8-Mo6** obtained from single crystal X-ray diffraction analysis. The C and H atoms were omitted for easy seeing.

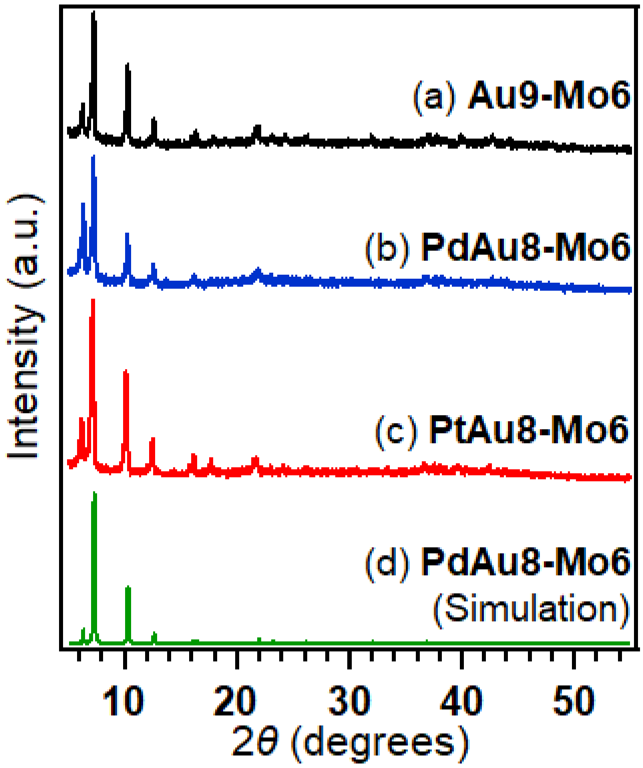

Figure. S14. Powder XRD patterns of (a) **Au9-Mo6**, (b) **PdAu8-Mo6** and (c) **PtAu8-Mo6**, and (d) simulated XRD pattern of **PdAu8-Mo6** obtained from single crystal X-ray diffraction analysis as shown in Fig. S13.

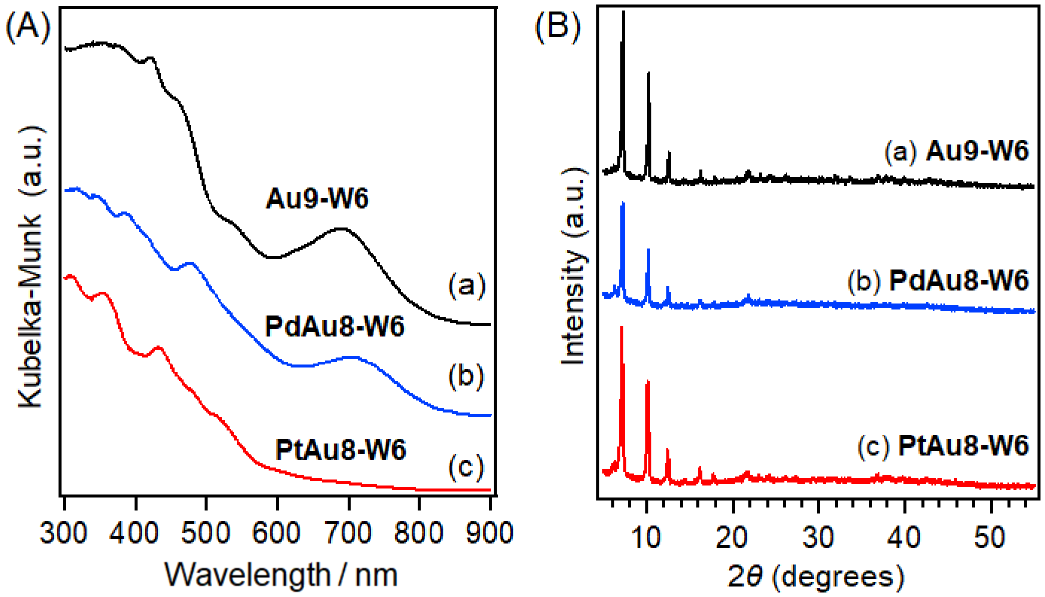

Figure. S15. (A) DR-UV-Vis-NIR spectra and (B) XRD patterns of (a) **Au9-W6**, (b) **PdAu8-W6**, and (c) **PtAu8-W6**.

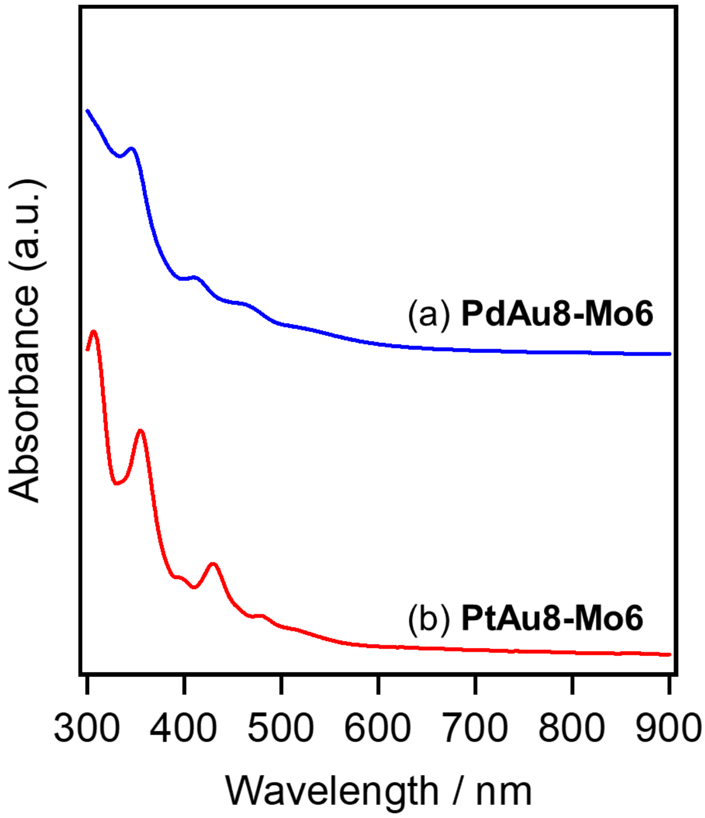

Figure. S16. UV-Vis-NIR spectra of (a) **PdAu8-Mo6** and (b) **PtAu8-Mo6** in DMSO.

Table S1. Results of curve fitting of Pd K-edge FT-EXAFS for **PdAu8-NO3**, **PdAu8-PMo12**, and **PdAu8-Mo6** and of Pt L_3-edge FT-EXAFS for **PtAu8-NO3**, **PtAu8-PMo12**, and **PtAu8-Mo6**.

**Edge**	**Sample**	**Bond**	**CN**	***r* / Å**	**σ^2**	**R-factor (%)**	**θ_E (K)**
Pd K	**PdAu8-NO3**	Pd–Au	7.4(2)	2.64(1)	0.06(1)	5.3	
Pd K	**PdAu8-PMo12**	Pd–Au	7.8(1)	2.63(1)	0.05(1)	6.5	212(9)
Pd K	**PdAu8-Mo6**	Pd–Au	7.8(2)	2.64(1)	0.06(1)	5.1	
Pt L_3	**PtAu8-NO3**	Pt–Au	8.0(4)	2.64(3)	0.06(3)	10.9	
Pt L_3	**PtAu8-PMo12**	Pt–Au	7.9(3)	2.64(3)	0.05(4)	7.5	207(40)
Pt L_3	**PtAu8-Mo6**	Pt–Au	8.1(3)	2.63(3)	0.05(3)	10.9	
Au L_3	**PdAu8-NO3**	Au–P	1.1(2)	2.28(4)	0.05(4)		
		Au–Pd	0.9(2)	2.64(3)	0.06(3)	8.1	
		Au–Au	2.0(2)	2.79(3)	0.07(2)		
Au L_3	**PtAu8-NO3**	Au–P	1.1(2)	2.22(4)	0.05(3)		
		Au–Pt	0.9(1)	2.66(2)	0.04(2)	11.6	
		Au–Au	2.1(2)	2.79(3)	0.07(2)		

CN: Coordination number; *r*: bond distance; σ^2: Debye-Waller factor. Numbers in parentheses represent uncertainties. The reliability factor (R-factor) is defined as:

R-factor $= \{\Sigma[k^3 \chi_{obs}(k) - k^3 \chi_{cal}(k)]^2 / \Sigma[k^3 \chi_{obs}(k)]^2\}^{1/2}$

where, χ_{obs} and χ_{cal} correspond to the observed and calculated data, respectively.

Table S2. Structural parameters of **Au9-C**, **Au9-B**, **PdAu8-C**, **PdAu8-B**, **PtAu8-C**, and **PtAu8-B** obtained by single-crystal (SC) XRD and DFT calculations

Structure	Method	Edge	Bond	CN	*r* / Å
**Au9-C**	SC-XRD[a]	Au L_3	Au–P	0.9	2.27
			Radial Au–Au	1.8	2.67
			Lateral Au–Au	1.8	2.79
	DFT[d]	Au L_3	Au–P	0.9	2.41 [2.28]
			Radial Au–Au	1.8	2.8 [2.65]
			Lateral Au–Au	1.8	2.96 [2.80]
**Au9-B**	SC-XRD[b]	Au L_3	Au–P	0.9	2.30
			Radial Au–Au	1.8	2.70
			Lateral Au–Au	2.7	2.83
	DFT[d]	Au L_3	Au–P	0.9	2.42 [2.29]
			Radial Au–Au	1.8	2.85 [2.70]
			Lateral Au–Au	2.7	2.99 [2.83]
**PdAu8-C**	SC-XRD[c]	Au L_3	Au–P	1.0	2.29
			Au–Pd	1.0	2.63
			Au–Au	2.0	2.80
		Pd K	Pd–Au	8.0	2.63
	DFT[d]	Au L_3	Au–P	1.0	2.42 [2.29]
			Au–Pd	1.0	2.74 [2.59]
			Au–Au	2.0	2.93 [2.77]
		Pd K	Pd–Au	8.0	2.74 [2.59]
**PdAu8-B**	DFT[d]	Au L_3	Au–P	1.0	2.48 [2.35]
			Au–Pd	1.0	2.8 [2.65]
			Au–Au	3.0	2.95 [2.79]
		Pd K	Pd–Au	8.0	2.8 [2.65]
**PtAu8-C**	SC-XRD[c]	Au L_3	Au–P	1.0	2.28
			Au–Pt	1.0	2.64
			Au–Au	2.0	2.82
		Pt L_3	Pt–Au	8.0	2.64
	DFT[d]	Au L_3	Au–P	1.0	2.41 [2.28]
			Au–Pt	1.0	2.74 [2.59]
			Au–Au	2.0	2.96 [2.80]
		Pt L_3	Pt–Au	8.0	2.74 [2.59]
**PtAu8-B**	DFT[d]	Au L_3	Au–P	1.0	2.43 [2.30]
			Au–Pt	1.0	2.79 [2.64]
			Au–Au	3.0	2.96 [2.80]
		Pt L_3	Pt–Au	8.0	2.79 [2.64]

a: M. Schulz-Dobrick *et al.*, *Eur. J. Inorg. Chem.*, **2006**, 4498 (2006).
b: F. Wen *et al.*, *Eur. J. Inorg. Chem.*, **2008**, 106 (2008).
c: T. Matsuyama *et al.*, *J. Chem. Phys.*, **155**, 044307 (2021).
d: Bond lengths determined by the average bond lengths for the model structures. Numbers in brackets are bond lengths reduced to 94.6% of their original values.

Table S3. Au L_3-edge EXAFS curve fitting results for **Au9-NO3**, **Au9-PMo12**, and **Au9-Mo6**.

Sample	Bond	CN	*r* / Å	σ^2	R-factor (%)	θ_E (K)
	Au–P	0.9(2)	2.34(4)	0.04(4)		
**Au9–NO3**	Au–Au1	1.1(1)	2.67(2)	0.04(2)	13.0	
	Au–Au2	3.0(3)	2.80(3)	0.08(2)		
	Au–P	1.0(2)	2.28(4)	0.05(4)		414(84)
**Au9–PMo12**	Au–Au1	1.0(1)	2.67(2)	0.04(2)	13.7	181(11)
	Au–Au2	2.1(2)	2.77(3)	0.07(2)		119(14)
	Au–P	1.0(2)	2.29(4)	0.05(4)		
**Au9–Mo6**	Au–Au1	1.0(1)	2.68(2)	0.04(2)	12.2	
	Au–Au2	3.0(4)	2.80(5)	0.10(3)		

CN: Coordination number; *r*: bond distance; σ^2: Debye-Waller factor. Numbers in parentheses represent uncertainties. The reliability factor (R-factor) is defined as:

R-factor $= \{\Sigma[k^3 \chi_{obs}(k) - k^3 \chi_{cal}(k)]^2 / \Sigma[k^3 \chi_{obs}(k)]^2\}^{1/2}$

where, χ_{obs} and χ_{cal} correspond to the observed and calculated data, respectively.

References

1. F. Wen *et al*., *Eur. J. Inorg. Chem.*, **2008**, 106 (2008).

2. T. Matsuyama *et al*., *J. Chem. Phys.*, **155**, 044307 (2021).
